# Bioactive Peptides and Probiotic Bacteria: Modulators of Human Health

**DOI:** 10.3390/foods15010045

**Published:** 2025-12-23

**Authors:** Qi Wang, Yafeng Zheng

**Affiliations:** 1 Institute of Food Science and Technology, Fujian Academy of Agricultural Sciences, Fuzhou 350003, China; 2 College of Food Science, Fujian Agriculture and Forestry University, Fuzhou 350002, China; zyffst@163.com

The global functional food market is experiencing an unprecedented expansion, driven by escalating consumer demand for dietary solutions that deliver health benefits beyond basic nutrition [1]. This surge reflects a fundamental paradigm shift from reactive disease treatment to proactive wellness management, where food is increasingly recognized as a primary modulator in physiological homeostasis and chronic disease prevention [2]. Contemporary consumers show heightened health literacy, demanding clean-label products with scientifically substantiated efficacy, natural origins, and minimal processing—preferences that compel innovation in bioactive ingredient discovery, sustainable production, and advanced delivery systems [3,4].

Fermentation technology has re-emerged as a cornerstone of modern functional food development, offering sustainable and efficient biotransformation pathways that simultaneously enhance nutritional value, safety, and sensory acceptability [5,6]. The strategic application of lactic acid bacteria (LAB), bifidobacteria, and tailored yeast consortia enables the production of bioactive metabolites—including peptides, polyphenols, and oligosaccharides—while mitigating antinutritional factors and extending product shelf life [7,8,9]. Recent breakthroughs in precision fermentation and synthetic biology have catalyzed a revolution in this space, transforming microbial cell factories into efficient biomanufacturing platforms for the sustainable production of high-value compounds, while substantially reducing environmental footprints [10]. This microbial mastery aligns seamlessly with circular economy principles, valorizing underutilized agricultural by-products and marine resources into premium functional ingredients.

Central to this evolving landscape are bioactive peptides and probiotics, which function synergistically through the gut microbiome to modulate systemic health. Bioactive peptides, encrypted within dietary proteins, exhibit pleiotropic activities—including antihypertensive, antioxidant, immunomodulatory, and antidiabetic effects—upon liberation via enzymatic hydrolysis or probiotic fermentation [6,11]. Marine-derived proteins, particularly from fish processing waste and microalgal biomass, represent sustainable reservoirs of potent bioactive peptides targeting metabolic syndrome and cardiovascular risk [12]. Concurrently, probiotics operate as dynamic therapeutic agents, reshaping gut microbial ecology, fortifying intestinal barrier integrity, and producing signaling molecules such as short-chain fatty acids (SCFAs) and neurotransmitter analogs [13]. The emerging gut–peptide–probiotic axis exemplifies personalized nutrition potential, wherein strain-specific proteolytic systems release tailored peptide profiles that interact with host receptors and microbial consortia to influence metabolic and immune networks.

Accelerating these advances is the integration of multi-omics platforms and artificial intelligence (AI) in food innovation. Metagenomics, metabolomics, and peptidomics unravel complex microbe–host interactions, enabling the rational design of fermented products with optimized bioactivity [14]. AI-driven predictive modeling streamlines strain selection, fermentation optimization, and peptide structure–function correlation, reducing development timelines while enhancing efficacy [15]. Complementary advances in nanoencapsulation and colloidal delivery systems address persistent challenges of bioactive stability and bioavailability, ensuring targeted gastrointestinal release and enhanced physiological potency [16]. As regulatory frameworks evolve and consumer awareness deepens, the convergence of biotechnology, nutritional science, and digital innovation will define the next generation of evidence-based functional foods, delivering personalized health solutions through sustainable, scalable, and sensorially appealing products that meet modern demands.

Building upon these dynamic trends, this Special Issue showcases cutting-edge research that bridges fermentation engineering and bioactive peptide science to accelerate functional food innovation. The eight contributions (seven original research articles and one systematic review) collectively address critical challenges in novel fermentation strategies, peptide characterization, and translational applications, reflecting the interdisciplinary synergy among food science, microbiology, and molecular biochemistry.

## 1. Fermentation as a Catalyst for Quality and Nutrition

Fermentation, an age-old food processing technique, has been revitalized through modern microbiological research, enabling the precise modulation of food properties [7]. Two studies in this Special Issue demonstrate the transformative impact of microbial fermentation on fruit juices. Guan et al. (Contribution 1) investigated the fermentation of mulberry juice using *Lactiplantibacillus plantarum* BXM2, revealing that fermentation not only reduced pH from 4.15 to 3.19 and extended shelf life but also significantly enriched bioactive metabolites. Through widely targeted metabolomics, 359 differential non-volatile metabolites (238 upregulated, 121 downregulated) and 26 key flavor compounds were identified, with lipid metabolism and amino acid catabolism implicated as core pathways for flavor enhancement. Notably, bioactive substances such as indole-3-lactic acid, octadeca-9,12,15-trienoic acid, and di-/tri-peptides were elevated, conferring additional health benefits—consistent with recent findings that lactic acid bacteria (LAB) fermentation of fruit juices enhances phenolic profiles and functional potential [17].

Complementing this, Fan et al. (Contribution 4) explored *Lacticaseibacillus rhamnosus* FJG1530 fermentation of mango juice, showing reduced total sugar content, increased total flavonoids, and enhanced antioxidant activity (ABTS and DPPH radical scavenging) alongside improved inhibition of lipase and α-glucosidase. Untargeted metabolomics identified 592 significantly altered non-volatile compounds, with amino acid metabolism a key driver of nutritional improvement.

In cereal-based products, Huang et al. (Contribution 2) focused on oolong tea-fortified rice noodles fermented with *Weizmannia coagulans* PR06. Fermentation (3% and 5% inoculation) altered amylopectin chain distribution, increased starch short-range order, and strengthened tea polyphenol–protein–starch interactions, resulting in improved hardness, chewiness, and cooking quality (reduced broken strip rate and cooking loss). Importantly, in vitro starch digestibility was significantly delayed, with resistant starch (RS) content elevated—addressing global concerns about glycemic control, as fermented cereals have been shown to enhance mineral bioavailability and reduce phytate levels. These studies collectively underscore fermentation’s dual role in optimizing sensory attributes and enriching functional components, providing sustainable solutions to overcome the limitations of traditional food processing (e.g., short shelf life, nutritional homogeneity).

## 2. Bioactive Peptides: From Natural Sources to Targeted Health Benefits

Bioactive peptides, derived from enzymatic hydrolysis or microbial fermentation of food proteins, represent a promising class of functional ingredients due to their safety, bioavailability, and diverse physiological activities [12]. Three studies in this Special Issue focus on the identification and characterization of peptides with specific health-promoting properties. Qi et al. (Contribution 5) heterologously expressed a recombinant ginseng tetradecapeptide (7RS14α) in *Saccharomyces cerevisiae* using a multicopy tandem strategy. The peptide significantly enhanced glucose uptake in insulin-resistant 3T3-L1 adipocytes, likely via the modulation of lipid metabolism and insulin signaling cascades—mirroring recent research on soybean sprout peptides that alleviate obesity through PI3K-Akt pathway regulation. This highlights the potential of recombinant peptide production as a scalable approach to developing hypoglycemic functional foods. 

Yang et al. (Contribution 6) prepared low-molecular-weight donkey-hide gelatin peptides (LMW DHGPs) with a high iron-chelating capacity (249.98 µg/mg). Structural characterization revealed that LMW DHGP–iron complexes form via α-helix-mediated self-assembly, with Asn, Gly, Cys, and Lys as key chelating sites. These complexes exhibited superior iron solubility under simulated gastrointestinal conditions and dual functionality (iron supplementation + antioxidant activity), addressing global iron deficiency challenges. This builds on findings that food-derived peptides can enhance mineral bioavailability through chelation, offering a natural alternative to synthetic fortificants.

Zhang et al. (Contribution 7) isolated four novel angiotensin I-converting enzyme (ACE)-inhibitory peptides (FAGGP, FDGY, FHPGY, WADP) from *Flammulina velutipes* via double enzymatic hydrolysis. Molecular docking confirmed a strong affinity with ACE active sites (binding energy: −9.4 to −10.3 kcal/mol), and in vitro validation showed IC_50_ values ranging from 14.79 to 91.55 µM. This not only expands the range of sources of natural ACE-inhibitory peptides—filling the gap of underutilized edible fungi in this field (compared to common dairy or marine sources)—but also provides a basis for subsequent structure–activity relationship studies.

## 3. Mechanistic Insights and Translational Potential

A key strength of this Special Issue lies in its emphasis on unraveling the molecular mechanisms underlying functional improvements. De la Rosa González et al. (Contribution 3) conducted a systematic review on probiotic-mediated activation of the aryl hydrocarbon receptor (AhR) pathway, demonstrating that probiotics (e.g., *Lactobacillus*, *Bifidobacterium*) enhance gastrointestinal health via tryptophan metabolites (e.g., indole-3-lactic acid, indole-3-acetic acid) and short-chain fatty acids (SCFAs). In healthy populations, AhR activation promotes intestinal immune tolerance, while in gastrointestinal pathologies (e.g., colitis, celiac disease), it ameliorates inflammation through the upregulation of anti-inflammatory cytokines (e.g., IL-22)—supporting the critical role of probiotic–microbiota–host crosstalk in functional food efficacy. 

Metabolomics has emerged as a powerful tool in these studies, as exemplified by untargeted mass spectrometry approaches that track dynamic changes in fermented food metabolomes, identifying key metabolites linked to tricarboxylic acid cycles and amino acid metabolism. Such mechanistic insights are essential in translating laboratory-scale findings to industrial applications, as highlighted in recent editorials calling for enhanced nutrient profiles and sustainability in fermentation technology.

Collectively, the studies in this Special Issue showcase the versatility of fermentation and bioactive peptide technology in addressing critical challenges in food science—from improving shelf life and sensory quality to developing targeted nutritional interventions for metabolic disorders (e.g., diabetes, hypertension, iron deficiency). Future research should focus on scaling up these technologies for industrial applications, validating in vivo efficacy, and exploring synergistic effects of multi-strain fermentation or peptide combinations. Additionally, advances in analytical techniques (e.g., single-cell metabolomics, the in silico prediction of peptide activity) will accelerate the discovery and development of novel functional ingredients. 

This Special Issue not only summarizes the latest advancements in functional food research but also suggests new directions for interdisciplinary collaboration, ultimately contributing to the global goal of promoting human health through sustainable, nutrient-dense food systems. We extend our gratitude to all authors, reviewers, and editorial staff for their invaluable contributions, and we anticipate that these findings will serve as a foundation for future innovation in functional food development.

## Data Availability

Not applicable.
